# The Preventive Effects of Fermented and Germinated Foxtail Millet Whole Grain on Kidney Damage in a Diabetic Mouse Model

**DOI:** 10.3389/fnut.2022.940404

**Published:** 2022-06-16

**Authors:** Xia Liu, Bin Qiu, Wei Liu, Yuhan Zhang, Xianshu Wang, Xingang Li, Lingfei Li, Di Zhang

**Affiliations:** ^1^Medical Integration and Practice Center, Cheeloo College of Medicine, Shandong University, Jinan, China; ^2^Shandong Academy of Agricultural Sciences, Jinan, China; ^3^Jinan Microecological Biomedicine Shandong Laboratory, Jinan, China; ^4^Department of Neurosurgery, Qilu Hospital, Shandong University, Jinan, China; ^5^College of Food Science and Technology, Yunnan Agricultural University, Kunming, China

**Keywords:** foxtail millet, fermentation, germination, diabetic kidney disease, gut microbiota

## Abstract

Diabetic kidney disease (DKD) is an important complication of diabetes. The prevention of DKD can effectively reduce the mortality rate of diabetic patients and improve their quality of life. The present study examined the effects of fermented and germinated foxtail millet whole grain (FG-FM) on kidney lesions in a diabetic mouse model (Db/Db mice). The results proved that the FG-FM consumption significantly alleviated the kidney tissue damage in the diabetic mouse model. The transcriptome analysis of kidney tissues demonstrated that the overactivation of signaling pathways related to inflammation and immunity in the diabetic mouse model was significantly inhibited with the FG-FM intake. Moreover, the consumption of the FG-FM diet effectively elevated the bacterial diversity, increased the relative abundance of probiotics and decreased the relative abundance of previously reported DKD-related bacteria in the gut microbiota of diabetic mice. Our study confirmed foxtail millet as a potential source of functional food for the non-pharmacological intervention of DKD.

## Introduction

Diabetes mellitus (DM) is a chronic metabolic disease with a dramatically increased incidence globally in the past decades. Over one million deaths are directly attributed to DM each year (1), and the main factors responsible for DM-related mortality are diabetes complications (2). Diabetic kidney disease (DKD) is one of the major complications of diabetes and the leading cause of chronic kidney disease and 20%-40% of diabetic patients have combined diabetic kidney disease (3, 4). The population survey showed that kidney disease is an important risk factor accounting for the increased mortality in patients with type 2 diabetes (5). Although the progress in pathology research has facilitated the study of target drug discovery, there is still no ideal drug for DKD because the success rate of these compounds in clinical trials has been disappointingly low (6). Therefore, the development of non-pharmacological DKD intervention is of great significance for improving both length and quality of life in the diabetic population.

The growing evidence support that gut microbiota plays a pivotal role in the pathogenesis of DKD. As a complex ecosystem with 100 trillion microbes, the gut microbiota executes a wide range of important biological functions, including nutrient absorption and metabolism, vitamin production, regulation of development, resistance to pathogens, and maintenance of immune homeostasis (7–11). Significant changes in the gut microbiota have been confirmed in a variety of diseases, including obesity, DM, DKD, inflammatory bowel disease, cardiovascular disease, and cancer (12–14). The composition of gut microbiota in the DKD patients was significantly different from that of non-DKD diabetes patients (15). A marked expansion of bacterial communities at different classification levels, including *Proteobacteria, Selenomonadales, Neosynechococcus, Shigella, Escherichia coli*, etc., were found in DKD patients when compared to diabetic patients without kidney disease (15). Consequently, the gut microbiota dysbiosis induces intestinal barrier damage, prompts the translocation of endotoxin and other toxins, aggravates the system inflammatory levels, and finally contributes to kidney injury (16–18). Moreover, Cai *et al*. also reported that the proportion of short chain fatty acids (SCFAs)-producing bacteria in gut microbiota was decreased in DKD patients, and the oral supplement of SCFAs could improve kidney injury in the diabetic mouse model (19). These studies suggested that targeting the gut microbiota could be a novel strategy for the prevention of DKD.

Diet is a direct and effective factor affecting the gut microbiota (20). The foxtail millet (*Setaria italica*) is one of the major food crops in northern China, which is rich in dietary fiber, minerals, vitamins, and proteins (21–23). Previously studies have proved that foxtail millet has anti-inflammatory property and may prevent chronic diseases such as atherosclerosis and diabetes (24, 25). Moreover, animal studies proved that the intake of cereal bran effectively promotes beneficial bacteria and increases the production of SCFAs in gut microbiota (26). Thus, foxtail millet can serve as an important source of prebiotics. In our previous study, the effects of different pretreated foxtail millet cereal flour on an acute colitis mouse model with severe gut microbial dysbiosis were studied (20). The results showed that the fermented and germinated foxtail millet whole grain (FG-FM) cereal flour had the strongest prebiotic function and could almost completely restore the gut microbiota disorder in the colitis mouse model (20). Our findings coincide with studies that have shown that germination or fermentation can improve the prebiotic performance of whole grain (27–29).

Based on the fact that the gut microbiota plays an important role in the pathogenesis of DKD, as well as the powerful prebiotic properties of the FG-FM, we hypothesize that FG-FM has an impact on the prevention or treatment of DKD. In the current study, we tested the effects of fermented and germinated foxtail millet whole grain (FG-FM) intake on diabetes-related kidney injuries in genetically diabetic C57BL/KsJ-db/db (Db/Db) mice and explored its impact on gut microbiota. Our study provides a cost-effective non-pharmacological strategy for the prevention or treatment of DKD.

## Materials and Methods

### Preparation of Cereal Flour From Fermented and Germinated Foxtail Millet Whole Grain (FG-FM)

The foxtail millet seeds were provided by the Crop Institute of Shandong Academy of Agricultural Sciences. The FG-FM cereal flour used for the animal study was produced from foxtail millet seeds as described previously (20). In brief, foxtail millet seeds were soaked in tap water for 12 h at room temperature. After the water was drained, the seeds were left to sprout at room temperature for 24 h. The germinated seeds were dried, ground, and then passed through an 80-mesh sieve to produce germinated whole-grain foxtail millet flour. The germinated whole grain foxtail millet flour was mixed with water (1:2), cooked for 10 min in a 75 ± 5°C water bath, and then fermented with *Lactobacillus Plantarum* NBRC 15,891 (obtained from NITE Biological Resource Center) at 37°C. The resulting slurry was dried, ground, and then passed through an 80-mesh sieve to produce germinated and fermented foxtail millet whole grain (FG-FM) cereal flour used in this study. The prepared FG-FM cereal flour contained 58.54% starch, 10.87% protein, 2.85% fat, 19.41% dietary fiber,5.47% moisture, and 2.87% ash.

### Animal Diets

The FG-FM cereal flour diet was designed based on the AIN-93M standard rodent formula. The cereal flour diet contained 50% FG-FM cereal flour, and the remaining 50% was supplemented with standard nutrients according to the AIN-93M formula. The control diet in the study was a standard AIN-93M rodent diet. The animal diets were prepared by Nantong Troffe feed Technology Co., Ltd (Jiangsu, China), and the detailed compositions of experimental diets were listed in the Supplementary Table S1.

### Animal Experiment Design

The 8–10 weeks old male genetically diabetic C57BL/KSJ-db/db (Db/Db) mice and their non-diabetic littermates C57BL/KSJ-m+/+db (Db/m) were obtained from GemPharmatech Co. Ltd. (Nanjing, China). The mice were housed in a specific pathogen-free facility (12 h daylight cycle) with *ad libitum* access to food and water, and the body weights were recorded every week. After 2 weeks of acclimation, mice were assigned into three groups (10 mice/group) with two different genotypes and two dietary treatments: 1) Db/m mice fed a standard AIN-93M rodent diet (CTRL group); 2) Db/Db mice fed a standard AIN-93M rodent diet (Db-93M group); 3) Db/Db mice fed an FG-FM cereal flour-based diet (Db-FM). The animal protocol was approved by the Institutional Animal Care and Use Committees of the Qilu Hospital of Shandong University.

### Tissue Collection and Histopathological Analysis

After 8 weeks on a diet, the mice were anesthetized with isoflurane inhalation and then terminated by cervical dislocation. By opening the abdomen cavity, the large intestine was removed and placed on an ice plate. Then cecal content was collected, snap-frozen in liquid nitrogen, and stored at −80°C until further analysis. The kidneys were removed and weighed, one of the kidneys was snap-frozen in liquid nitrogen and stored at −80°C for further total RNA extraction and transcriptome analysis, the other was processed for paraffin embedding, cut into 5 μm sections, and finally stained with hematoxylin and eosin (H&E) for histopathological analysis. The pathological features of liver tissue were also analyzed by H&E staining. Other tissues were removed and weighed, including the spleen and epididymal fat.

### Analysis of Cecal Microbiota Composition by 16s Ribosomal RNA (16s rRNA) Gene Sequencing

Total bacterial DNA was extracted from frozen cecal contents using a QIAamp DNA Stool Mini Kit (Qiagen, Valencia, CA). The region V3–V4 of the 16S rRNA gene was amplified, purified, and quantified sequentially. Then the DNA libraries were constructed following the manufacturer's instructions. After quality inspection, the constructed DNA library was sequenced with Illumina HiSeq 2,500 platform (Illumina, Inc, San Diego, California). The resulting pair-end reads were joined by fastq-join (Version 1.3.1, https://code.google.com/p/ea-utils/) and pear (30), and then cut and quality filtered by Cutadapt (version 1.18) (31) to obtain clean tags. After that, the resulting clean tags were assigned to OTUs using USEARCH (Version 11.0.667, http://www.drive5.com/usearch/) with a 97% threshold of pairwise identity. The OTUs were then aligned against the Silva database (Release132, http://www.arb-silva.de) (32). QIIME (33) software was used to generate an information table of the relative abundance of bacterial communities at different classification levels, and then the R software was used to plot the community structure at each taxonomic level of the sample. The linear discriminant analysis (LDA) effect size (LEfSE, https://huttenhower.sph.harvard.edu/galaxy/) was applied to identify bacterial communities responsible for the differences in cecal microbiota compositions between different groups, using an LDA score threshold of >4.0. The raw Illumina read data were uploaded into SRA at NCBI under the BioProject ID PRJNA835687.

### RNA Sequencing of Kidney Tissue and Analysis of Transcriptome Profiling Data

Total RNA was extracted from frozen kidney tissue, and the concentration and quality of extracted total RNA were determined by Nanodrop 2000, agarose gel electrophoresis, and Agilent 2100 bioanalyzer (Agilent, Santa Clara, CA, USA) in order. After the mRNA was purified from total RNA using Oligo (dT) beads (NEB, San Diego, CA, USA). The Illumina TruseqTM RNA sample prep Kit was used for sequencing library preparation based on purified mRNA samples. The resulting library fragments were quantified using an Agilent High Sensitivity DNA assay on a Bioanalyzer 2100 system (Agilent, Santa Clara, CA, USA) for concentration and size distribution, and the molar concentration of DNA libraries was analyzed by q-PCR using KAPA SYBR FAST Universal 2X qPCR Master Mix and DNA Quantification Standards and Primer Premix Kit (KAPA Biosystems, Woburn, MA, USA). The prepared libraries were mixed proportionally, then sequenced using an Illumina Novaseq 6000 platform (read length 2 × 150 bp).

After adaptor removal, quality and size trimming, low complexity filtering, and ribosomal RNA (rRNA) removal of raw data, the resulting clean reads were mapped to the Mus musculus reference genome using the program HISAT2 (https://ccb.jhu.edu/software/hisat2/index.shtml) (34). After assessment of mapping results, the transcripts were assembled and annotated using Cufflinks (https://cole-trapnelllab.github.io/cufflinks/) (35) or StringTie (https://ccb.jhu.edu/software/stringtie/) (36). Then, read counts of annotated transcripts (genes) were calculated using RSEM (37), and then transformed into FPMK (fragments per kilobases per million fragments) values for further analysis. Differentially expressed genes (DEGs) were analyzed using DESeq2 or edgeR software (38, 39). GO (Gene Ontology) and KEGG (Kyoto Encyclopedia of Genes and Genomes) enrichment analysis of DEGs was performed with Goatools (https://github.com/tanghaibao/GOatools) (40) and R software.

### Statistical Analysis

One-way analysis of variance (ANOVA) followed by Fisher's LSD test (GraphPad Software, Inc., La Jolla, California) was used for multiple comparisons. The results were considered statistically significant when *p* < 0.05. Data were expressed as means ± SEM.

## Results

### Physiological Parameters of Mice

**Figure 1:**
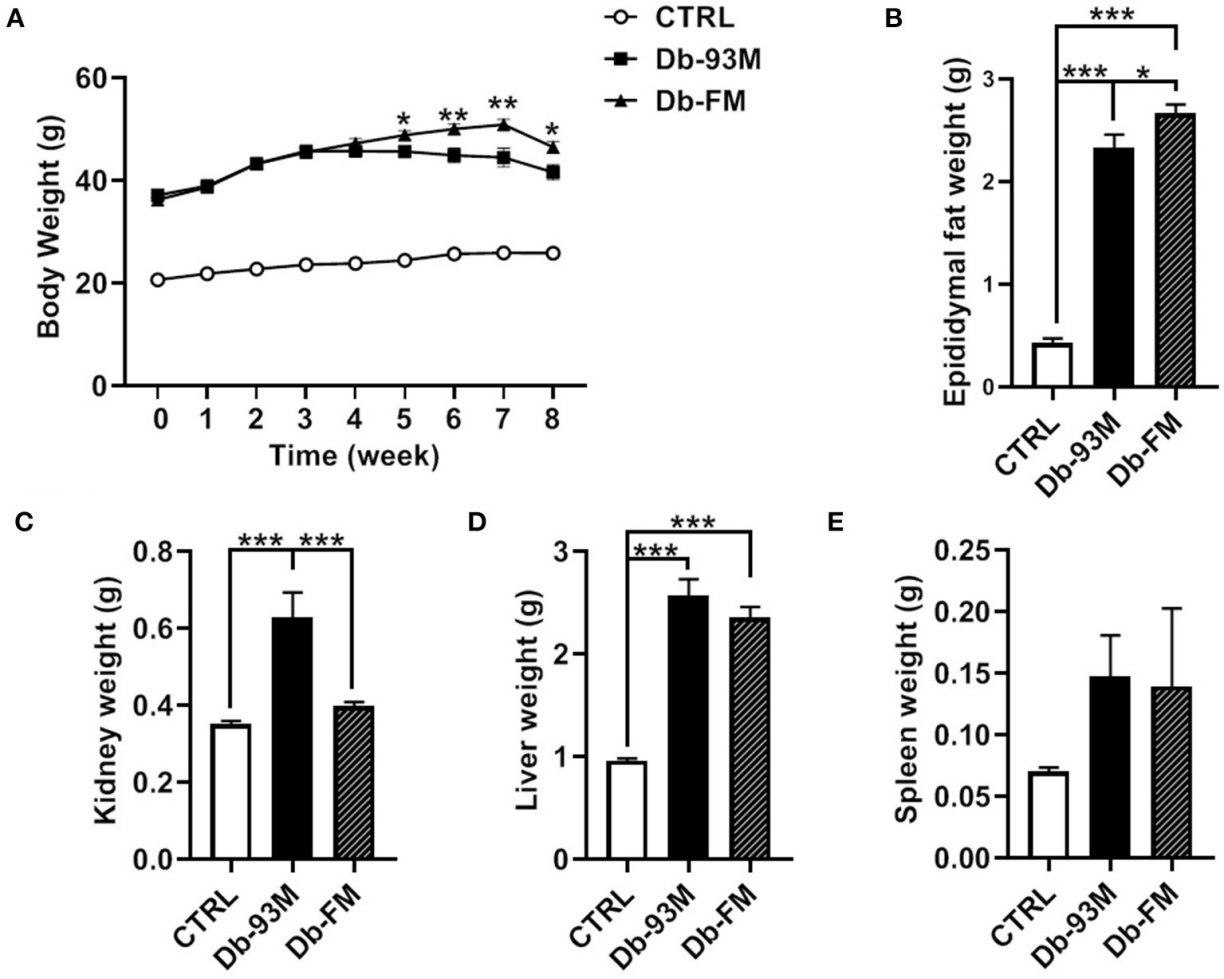
Physiological parameters. **(A)** Body weight changes during 8 weeks on diets, asterisks indicate significant differences between the Db-93M group and the Db-FM group (*p < 0.05, **p < 0.01). **(B)** Epididymal fat weights comparison. **(C)** Kidney weights comparison. **(D)** Liver weights comparison. **(E)** Spleen weights comparison. Data are expressed as the mean ± SEM.

As demonstrated in Figure 1A, the body weights of Db/Db mice (Db-93M group and Db-FM group) were significantly higher than that of Db/m mice (CTRL group) due to the genotypic difference. In the first 4 weeks on diets, the body weights of both groups of Db/Db mice (Db-93M and Db-FM) increased with no significant difference observed between groups. However, from the 5th week, the body weights of the mice in the Db-93M group began to decline, resulting in significantly lower body weights of the mice in the Db-93M group than those of mice in the Db-FM group. Consistent with body-weight loss, the epididymal fat weights of mice from the Db-93M group were significantly lower than that of the Db-FM group (Figure 1B, *p* = 0.0127). On the contrary, the kidney weights of mice from the Db-93M group were significantly higher than those from the Db-FM group (*p* < 0.0001, Figure 1C). Liver and kidney weights did not differ significantly between Db-93M and Db-FM groups.

### Histopathological Analysis

**Figure 2:**
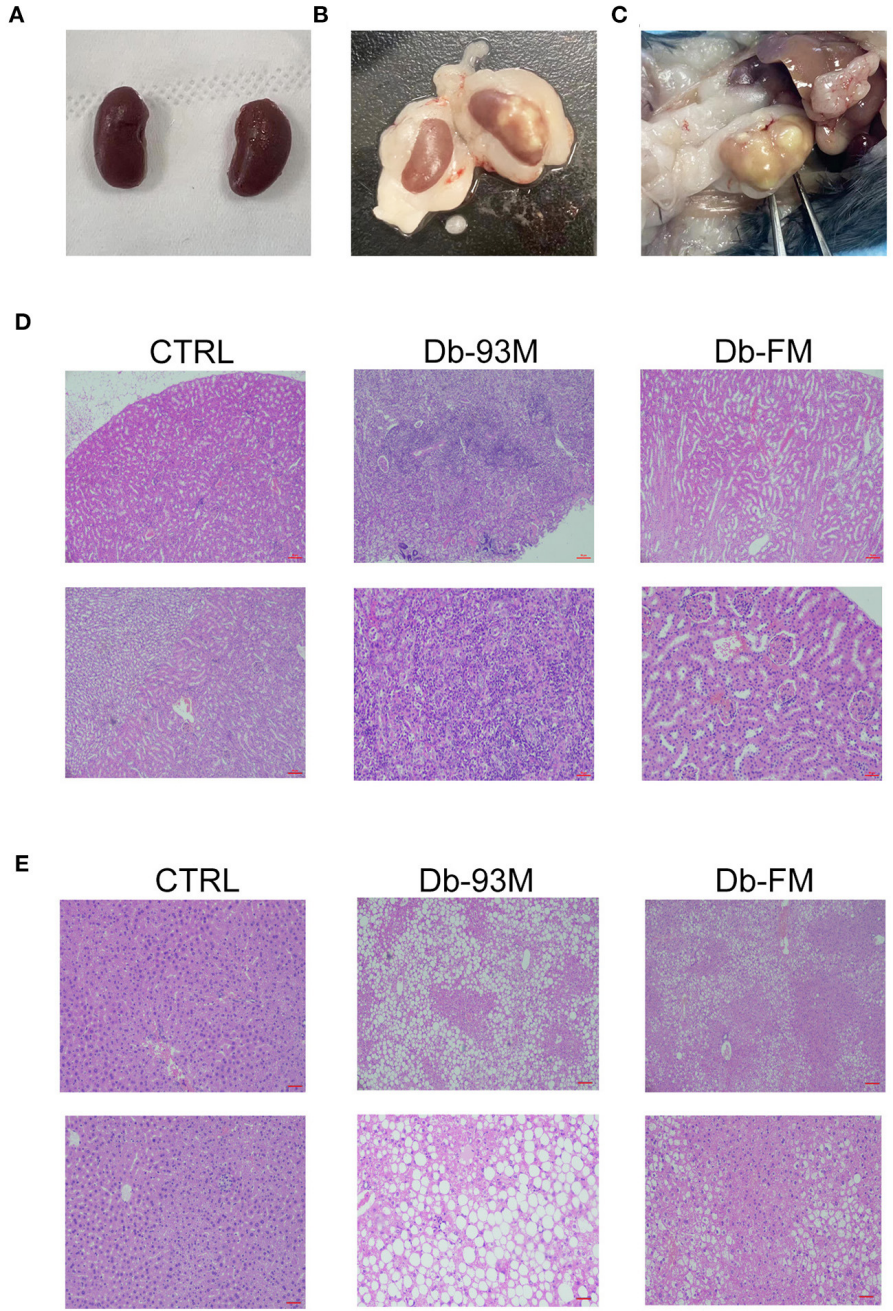
Histopathological analysis. **(A)** The appearance and morphology of the kidneys from CTRL group; **(B)** the appearance and morphology of the kidneys, the left one is the kidney from the Db-FM group, the right one is the kidney from the Db-93M group; **(C)** the kidney of mice from the Db-93M group; **(D)** histopathological examination of kidney tissues (top: 40 ×; bottom: 200 ×); **(E)** histopathological examination of liver tissues (top: 40 ×; bottom: 200 ×).

The experimental mice were executed after 8 weeks on diets. It was found that the kidneys in the CTRL group (Figure 2A) were normal in appearance and morphology while in both groups of Db mice, the kidney was surrounded by a large amount of fat. Six of the total nine mice in the Db-93M group had severe lesions in at least one of the kidneys (Figure 2B, right; Figure 2C), while the kidney of mice in the Db-FM group showed no morphological abnormalities (Figure 2B, left). The result of H&E staining (Figure 2D) clearly showed that the normal micro-structure of the kidney tissues was almost destroyed in the Db-93M group. In contrast, in the CTRL and Db-FM groups, the micro-structure of kidney tissues remained intact. These results together suggested that the FG-FM diet significantly alleviated kidney injury in the diabetic mouse model.

The kidney lesions of the mice in the Db-93M group were also associated with weight loss in the later period of feeding (from the 5th week onwards), due to the weight loss is an important feature of a mouse model with kidney disease. The Db/Db mice (Db-93M and Db-FM) had obvious fat droplets formation in the liver tissue compared to the CTRL group (Figure 2E).

### Comparative Transcriptome Analysis of Kidney Tissues

An average of 50.38 ± 0.31, 52.23 ± 0.40, and 50.72 ± 0.34 million clean reads were obtained for the kidney tissues in mice from CTRL, Db-93M, and Db-FM groups, respectively. The clean reads were mapped to the mouse reference genome (http://asia.ensembl.org/Mus_musculus/Info/Index) with high proportions: CTRL group, 96.41%; Db-93M group, 96.94%; and Db-FM group, 96.93%.

**Figure 3:**
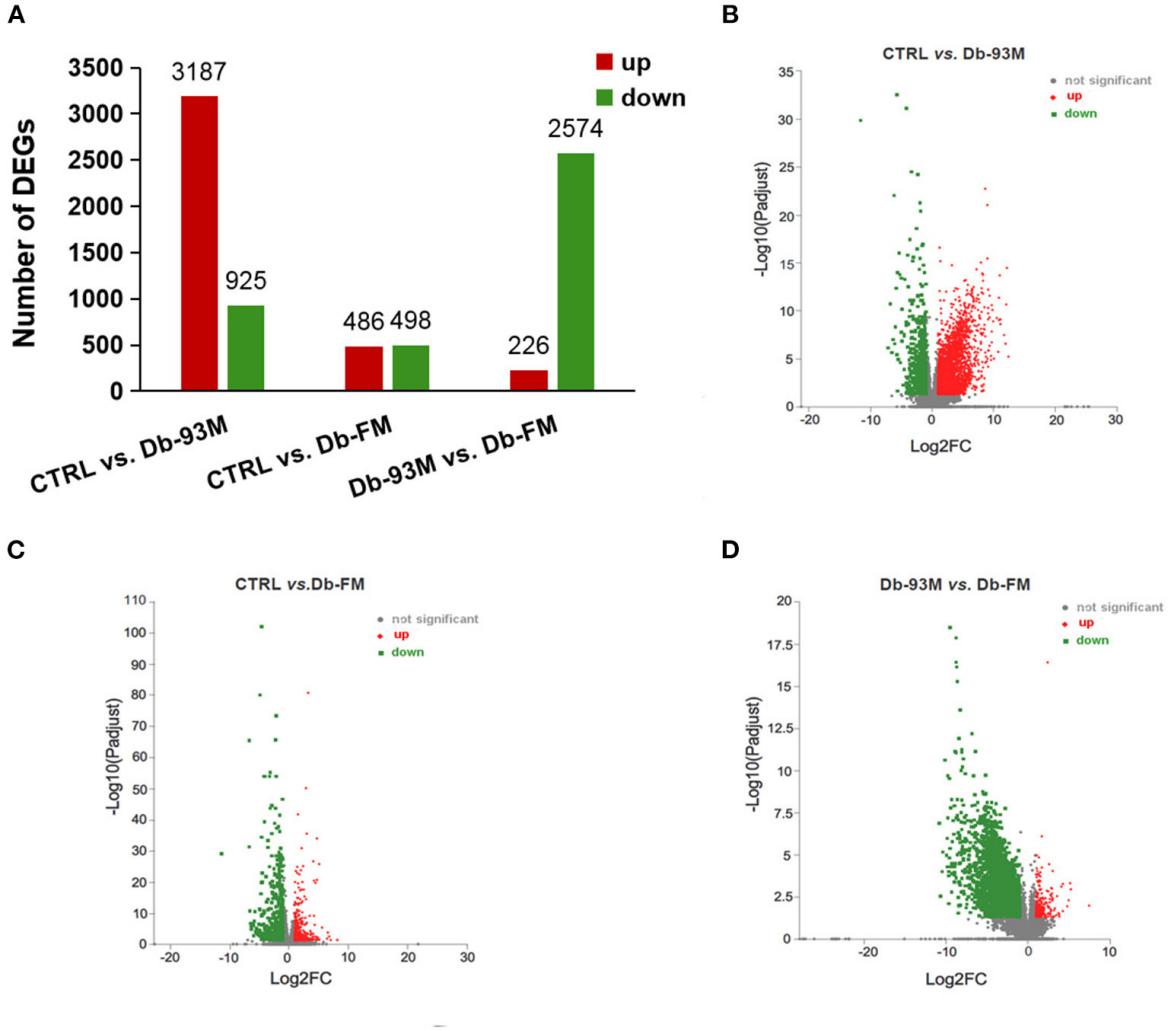
The gene expression analysis performed by RNA-sequencing (RNA-Seq, *n* = 6). **(A)** Numbers of differential expressed genes (DEGs) in kidney tissue of different groups; the volcanic maps for DEGs in **(B)** CTRL *vs*. Db-93M group; the red dots were up-regulated, and the green dots were down-regulated in Db-93M group when compared to CTRL group. **(C)** CTRL *vs*. Db-FM groups; the red dots were up-regulated, and the green dots were down-regulated in the Db-FM group when compared to the CTRL group. **(D)** Db-93M *vs*. Db-FM group; the red dots were up-regulated, and the green dots were down-regulated in the Db-FM group when compared to the Db-93M group.

The significantly differentially expressed genes (DEGs) were identified based on the quantification and comparison of gene expression levels of kidney tissues in mice from CTRL, Db-93M, and Db-FM groups. As shown in Figure 3, when comparing the groups with different genotypes fed with the same AIN-93M diet (CTRL *vs*. Db-93M), 4,112 DEGs, including 3,187 upregulated and 925 downregulated genes, were identified. When comparing the Db/Db groups fed with different diets (Db-93M *vs*. Db-FM), 2,800 DEGs, including 226 upregulated and 2,574 downregulated genes, were identified. Surprisingly, two groups of mice with different genotypes and different feeding (CTRL *vs*. Db-FM) showed minimal differences. Only 984 DEGs were identified, including 486 upregulated and 498 downregulated genes.

**Figure 4:**
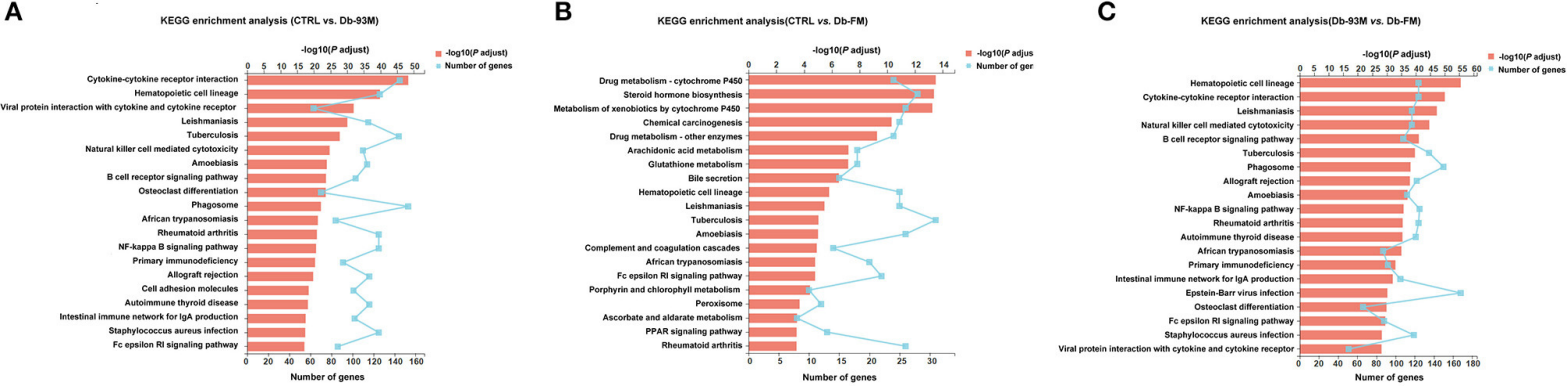
The KEGG enrichment results (kidney tissues) based on the identified DEGs from. **(A)** Comparison of CTRL group and Db-93M group. **(B)** Comparison of CTRL group and Db-FM group. **(C)** Comparison of Db-93M group and Db-FM group.

The KEGG enrichment analyses were performed to identify biological pathways significantly affected by different treatments. As shown in Figure 4A, when comparing the CTRL group and the Db-93M group, several signaling pathways related to inflammation, infection, and immunity were affected, including cytokine-cytokine receptor interaction pathway, natural killer mediated cytotoxicity pathway, B cell receptor signaling pathway, NF-κB signaling pathway, *etc*. The comparison between the Db-93M group and the Db-FM group also showed similar characteristics (Figure 4C). However, the affected signaling pathways in the comparison between the CTRL group and the Db-FM group did not show obvious associations with inflammation and immunity biological process (Figure 4B). Furthermore, among the top 20 most significant pathways resulting from KEGG enrichment analysis, 15 signaling pathways contained more than 100 DEGs in the comparison between CTRL and Db-93M, and also 15 in the comparison between Db-93M and Db-FM. Notably, none of the signaling pathways had more than 100 DEGs in the comparison between CTRL and Db-FM, and even the most affected signaling pathway contained only 36 DEGs. This also proved that, from the perspective of the transcriptome, the difference between the CTRL group and the Db-FM group was relatively inconspicuous, while the Db-93M group was significantly different from the other two groups.

When further analyzing the details of DEGs, a clear trend was observed. The changes that occurred in the Db-93M group (compared to the CTRL group) could be corrected in the Db-FM group. Take the cytokine-cytokine receptor interaction pathway as an example (Supplementary Figure S1), the abundance of chemokines and their receptors, including CCLs and CXCLs, as well as inflammatory cytokines and corresponding receptors, were significantly elevated in the Db-93M group when compared to CTRL group (Supplementary Figure S1A). Simultaneously, most of these upregulated genes were found to be significantly downregulated in the Db-FM group, when compared to the Db-93M group (Supplementary Figure S1B). Subsequently, the overactivation of the downstream signaling pathway, such as the NF-κB signaling pathway caused by the overexpression of chemokines and inflammatory cytokines in the Db-93M group was also normalized in the Db-FM group (Supplementary Figure S2). In addition, other signaling pathways related to inflammation and immunity also showed a similar trend, which was overactivated in Db-93M, and this overactivation was re-inhibited in the Db-FM group (Supplementary Figures S3, S4, and S5). Taken together, these results showed that intake of FG-FM could significantly alleviate kidney damage in diabetic mice by inhibiting the overactivation of signaling pathways in inflammation, infection, or immunity in the kidney.

### The Gut Microbiota Composition Analysis

The microbial species richness (Chao, Supplementary Figure 5A) and diversity (Shannon, Supplementary Figure 5B) index were calculated to analyze the effect of FG-FM on the gut microbiota of Db/Db mice. There was no significant difference in the Chao index among the three groups, indicating that neither the genotypic difference nor FG-FM had a significant effect on the bacterial species richness of the gut microbiota. Moreover, no significant variations in the species diversity between the CTRL group and Db/Db groups were evidenced by the Shannon diversity index (*p* = 0.17). However, the Shannon index of Db-FM mice was significantly higher than that of Db-93M mice (*p* = 0.0061), indicating that FG-FM significantly enhanced the bacterial species diversity of gut microbiota in Db/Db mice.

**Figure 5:**
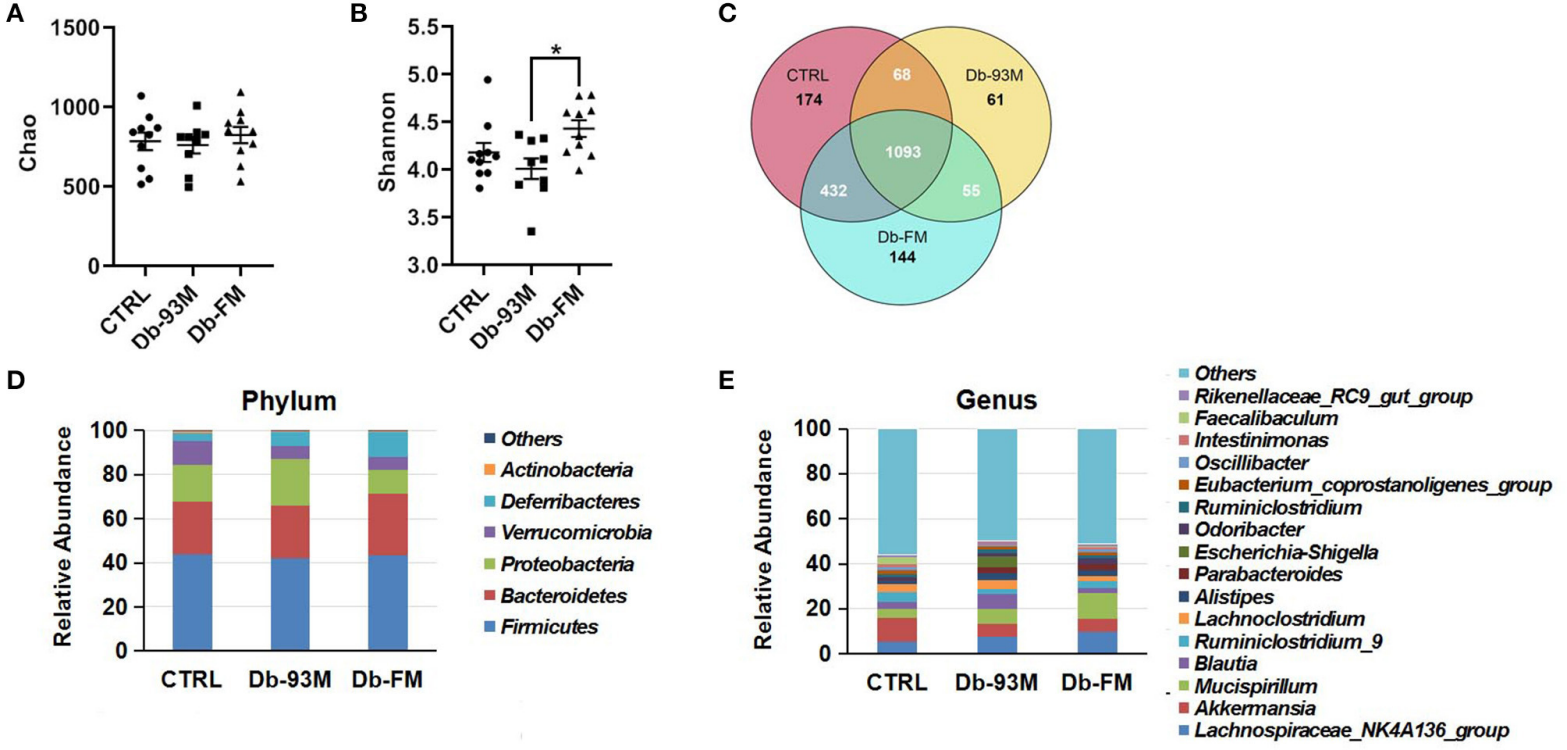
The gut microbiota composition analysis. **(A)** The microbial richness index of Chao. **(B)** The microbial diversity index of Shannon. **(C)** Venn diagrams that illustrated observed overlap of OTUs from different groups. **(D)** The composition of gut microbiota in mice at phylum level. **(E)** The composition of gut microbiota in mice at the genus level. “Others” represent the sum of all the phyla/genera, which abundances were under 1%. Data are expressed as the mean ± SEM.

The shared and specific OTUs analysis was demonstrated by the Venn diagram in Figure 5C. The gut microbiota of mice from the CTRL group had the largest number of unique OTUs (174), followed by that of the Db-FM group (144). Moreover, except for the OTUs shared by three groups (1093), the CTRL group and Db-FM had 432 shared OTUs, while the Db-93M group only shared 68 OTUs with the CTRL group and 55 OTUs with the Db-FM group. From these results, the intake of FG-FM could effectively change the gut microbiota of Db/Db mice, making them have more common microbiota characteristics with the CTRL group.

As illustrated in Figures 5D,E, gut microbiota composition varied in different groups. At the phylum level, the most abundant bacterial taxa was *Firmicutes* (CTRL, 43.90%; Db-93M, 41.93%; Db-FM, 43.60%) in all the three groups. The second dominant phylum was *Bacteroidetes* in gut microbiota (CTRL, 23.61%; Db-93M, 23.78%; Db-FM, 27.66%). Whereas in the Db-93M group, the relative abundance of phylum *Proteobacteria* (21.52%) increased dramatically, far more than the proportion of *Proteobacteria* in the gut microbiota from the other two groups (CTRL, 16.83%; Db-FM, 10.70%). At the genus level, the most abundant bacterial taxa in the CTRL group was *Akkermansia* (CTRL, 10.59%), followed by the *Lachnospiraceae* NK4A136 group (5.42%); in the Db-93M group, the most abundant bacterial taxa was *Lachnospiraceae* NK4A136 group (7.78%), followed by genus *Blautia* (6.58%); in Db-FM group, the most abundant bacterial taxa was *Mucispirillum* (11.32%), followed by genus *Lachnospiraceae* NK4A136 group (9.90%).

Taken together, these results showed that the consumption of FG-FM could effectively increase the bacterial diversity and reduce the proportion of phylum *Proteobacteria* in the gut microbiota of Db/Db mice. These prebiotic properties may contribute to preventing kidney disease in the diabetic mouse model.

### LEfSe Analysis of Gut Microbiota

**Figure 6:**
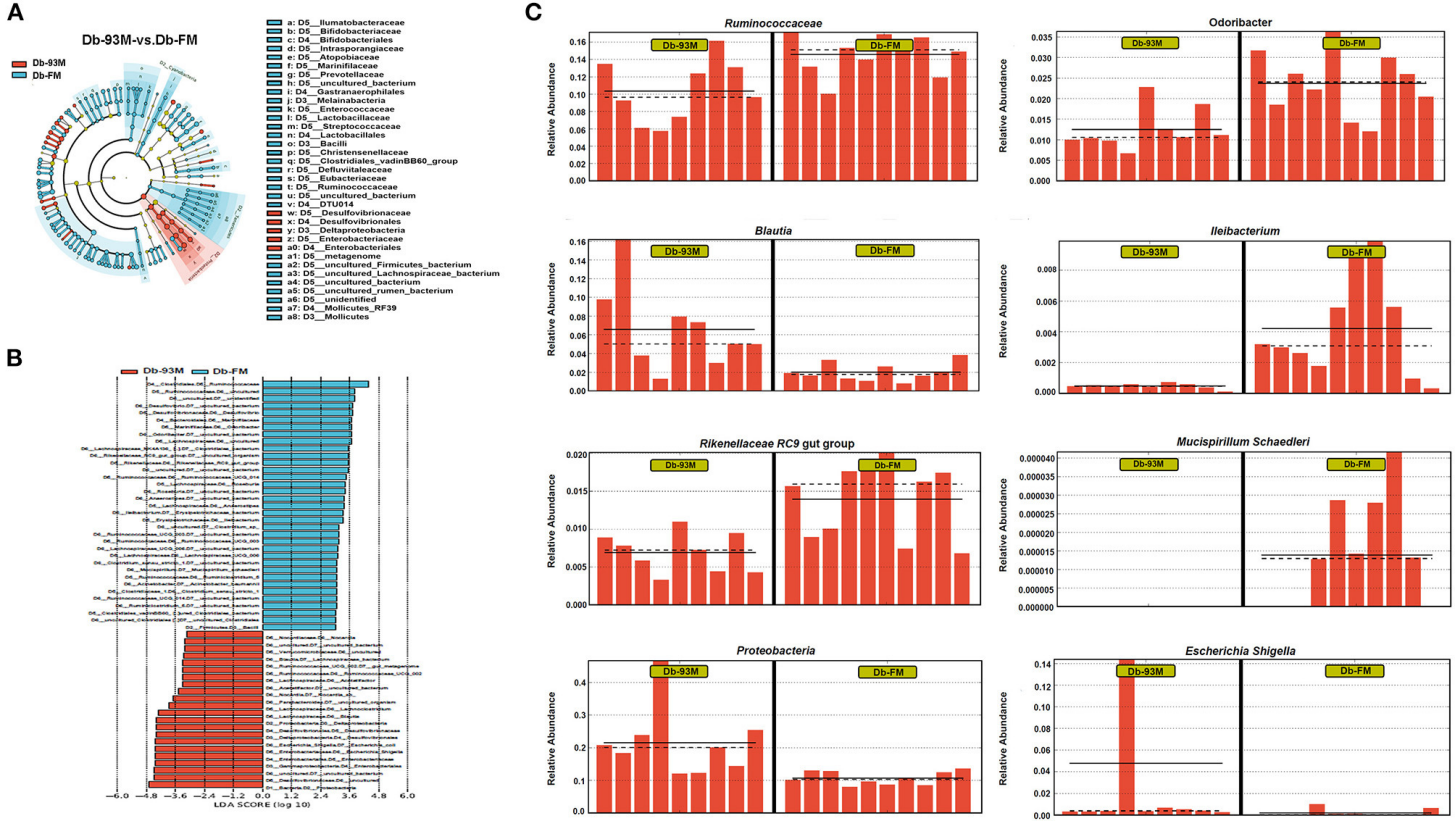
LEfSe analysis of gut microbiota. **(A)** Effect size of significantly enriched taxa in each group when performing a comparison between Db-93M group and Db-FM group. **(B)** Taxonomic cladogram obtained using LEfSe analysis result from the comparison between Db-93Mgroup and Db-FM group. **(C)** Relative abundance of representative bacterial taxa in the gut microbiota of Db-93M group and Db-FM group identified by LEfSe analysis.

The effects of FG-FM on the gut microbiota of Db/Db mice were further analyzed by LFfSe (LDA Effect Size), and the results (LEfSe Cladogram and histogram of LDA scores) were demonstrated in Figures 6A,B. By comparing the gut microbiota of the Db-93M and Db-FM groups, a total of 57 different bacterial taxa were found, including 22 dominant bacterial taxa in the gut microbiota of the Db-FM group and 35 dominant bacterial taxa in the Db-93M group. The relative abundances of several commensal bacteria that have been reported contributing to inflammation control and disease prevention were significantly higher in the Db-FM group than in the Db-93M group. For example, *Ruminococcaceae* (41), *Odoribacter* (42), *Ileibacterium* (43), *Lachnospiraceae* NK4A136 (44), *Rikenellaceae* RC9 gut group (45), and *Mucispirillum schaedleri* (46). The details of representative taxa are shown in Figure 6C. Furthermore, the important “signature” bacteria of dysbiosis in gut microbiota, including *Proteobacteria* and *Escherichia-Shigella* (47–49) were found to be significantly enriched in the Db-93M group. The results of the LEfSe analysis further validated the prebiotic characteristics of FG-FM cereal flour, including promoting the proliferation of probiotics and limiting the expansion of previously reported DKD-related bacteria in the gut microbiota of diabetic mouse model (15).

## Discussion

A substantial proportion of diabetic patients will develop DKD, which is the leading cause of chronic kidney disease and even end-stage kidney disease globally (50). Dietary and nutritional factors play key roles in the progression of DKD (51, 52). Foxtail millet is a typical underutilized crop which has received less attention than main crops. In the current study, we have identified a novel function of FG-FM in preventing kidney damage associated with diabetes mellitus. The FG-FM diet significantly alleviated the morphological lesions of the kidney as well as the overactivation of inflammatory and cytokine signaling pathways in the kidney of diabetic mice. Moreover, the gut microbiota analysis revealed the FG-FM diet as an excellent prebiotic in the prevention of kidney disease. Our study provided a novel non-pharmacological intervention strategy for Diabetic kidney disease (DKD)

Transcriptomics analysis revealed that a large number of genes involved in inflammatory and immune signaling pathways were overactivated in the kidney tissues of Db-93M mice, indicating a high level of inflammation in the kidney of diabetic mice. In addition, we also observed severe kidney lesions in these mice when no special diet intervention was applied. These results clearly demonstrated the development of DKD in diabetic mice. However, the FG-FM intervention protected the kidney from developing both the high levels of inflammation and the morphological damage in the diabetic mice. Inflammation is a well-established feature of DKD, manifested as the increased level of inflammatory cytokines in the serum of DKD patients, as well as anti-inflammatory treatments are effective in the prevention of DKD (53, 54). Notably, we found that the transcription of chemokines and their receptors, including CCLs and CXCLs, as well as several inflammatory cytokines, such as IL1β and TNFα, were significantly upregulated in the kidney of diabetic mice, while such elevation was not observed in mice fed with the FG-FM diet. In support of our results, the overexpression of CCLs, CXCLs, and inflammatory cytokines has been proved to be associated with DKD or other kidney diseases (55–57) and has been widely used to induce kidney injury in animal models (58–60). Besides chemokines, the over expression of several inflammatory cytokines, such as IL1β and TNFα, have been identified as biomarkers in diabetic kidney disease (61). Their overexpression is not only an indicator of kidney inflammation but also a trigger for further deterioration of renal lesions (62). Therefore, the suppression of these hyperactivated inflammatory or immune-related cytokines and signaling pathways in the Db-FM group could strongly explain the rescue effects of FG-FM on kidney lesions in diabetic mice. Our findings not only confirmed the function of the FG-FM diet at the transcriptional level, but also partially explained its molecular mechanism for relieving renal lesions.

Consistent with the alleviated DKD symptoms in Db-FM groups, improvement in the gut microbiota was also identified. In line with what we found, the reduction of bacterial diversity has been reported previously exist in patients (63, 64) and animal models (65) with kidney disease. We, therefore, speculate that the promotion of microbial diversity index may be attributed to the prebiotic properties of FG-FM and beneficial for the alleviation of nephropathy symptoms in diabetic mice (66). The proliferation of probiotics in the gut microbiota of the Db-FM group is also important evidence of the prebiotic function of FG-FM. For example, the relative abundance of several SCFAs producing bacteria, such as *Odoribacter, Lachnospiraceae NK4A136*, and *Rikenellaceae RC9 gut group* (67–69), was obviously increased by FG-FM ingestion. SCFAs have the ability to ameliorate diabetic nephropathy via inhibition of the NF-κB signaling pathway (70). Coincidentally, the transcriptome analysis displayed that the overactivation of the NF-κB signaling pathway in the Db-93M group was normalized in the Db-FM group. Furthermore, the unusual expansion of *Proteobacteria* and *Escherichia-Shigella* has been considered as common features of the imbalanced gut microbiota in different inflammatory-related diseases (49, 71, 72), and the decrease in the proportion of *Proteobacteria* and *Escherichia-Shigella* in the gut microbiota of mice in the Db-FM group could be regarded as a sign of the improved gut microbiota. Taken together, our study proved that FG-FM could inhibit the overactivation of inflammatory signaling pathways via restoring the pro-inflammatory characteristics of gut microbiota, thereby preventing the occurrence of renal lesions in diabetic mice.

In conclusion, our study demonstrated that diet intervention with fermented and germinated foxtail millet whole grain (FG-FM) could effectively prevent and protect DKD in the diabetic mouse model. At the same time, we revealed the excellent prebiotic function of FG-FM in preventing kidney disease. The promising results from our animal study provide a non-pharmacological strategy for preventing DKD through functional foods developed based on foxtail millet whole grain.

## Data Availability Statement

The datasets presented in this study can be found in online repositories. The names of the repository/repositories and accession number(s) can be found below: NCBI SRA; PRJNA835687.

## Ethics Statement

The animal study was reviewed and approved by Institutional Animal Care and Use Committees of the Qilu Hospital of Shandong University.

## Author Contributions

Conceptualization, data curation, and validation: DZ and LL. Methodology: XLiu, WL, and BQ. Investigation: XLiu, WL, BQ, YZ, and XW. Writing-original draft preparation: XLiu and WL. Writing-review and editing: DZ and XLi. Project administration: DZ. Funding acquisition: DZ and BQ. All authors have read and agreed to the published version of the manuscript.

## Funding

This research was funded by National Science Foundation of China grants (82071512), Shandong Provincial Natural Science Foundation grant (ZR2019ZD33), Shandong Provincial Key Research and Development program (2019GHZ031), and Research Project of Jinan Microecological Biomedicine Shandong Laboratory (JNL-2022003A to XLi).

## Conflict of Interest

The authors declare that the research was conducted in the absence of any commercial or financial relationships that could be construed as a potential conflict of interest.

## Publisher's Note

All claims expressed in this article are solely those of the authors and do not necessarily represent those of their affiliated organizations, or those of the publisher, the editors and the reviewers. Any product that may be evaluated in this article, or claim that may be made by its manufacturer, is not guaranteed or endorsed by the publisher.
