# Supplementary material for: The Preventive Effects of Fermented and Germinated Foxtail Millet Whole Grain on Kidney Damage in a Diabetic Mouse Model
Source: Front Nutr. 2022 Jun 16;9:940404. doi: 10.3389/fnut.2022.940404 (PMC9243661; doi:10.3389/fnut.2022.940404)
Supplement: Supplementary file 1 [file Data_Sheet_1.ZIP › Table S1.docx]

**Table S1: Composition of experimental diets used in this study**

| **Composition** | **Diet (g/1000 g)** | |
| --- | --- | --- |
|  | **AIN-93M** | **FG-FM cereal flour diet** |
| Casein | 140 | 85.65 |
| L-Cystine | 1.8 | 1.8 |
| Corn starch | 495.69 | 100.04 |
| foxtail millet cereal flour | 0 | 500 (FG-FM cereal flour) |
| Maltodextrin | 125 | 125 |
| Sucrose | 100 | 100 |
| Cellulose | 50 | 0 |
| Soybean Oil | 40 | 40 |
| Mineral mixture(AIN-93M-MX)* | 35 | 35 |
| Vitamin mixture(AIN-93-VX)* | 10 | 10 |
| Bitartrate Choline | 2.5 | 2.5 |
| Tert-butylhydroquinone | 0.008 | 0.008 |
| Total | 1000 | 1000 |

The compositions of mineral and vitamin mixture were followed the description of Reeves *et al.*, 1993.
