# Supplementary figures and images for: The Preventive Effects of Fermented and Germinated Foxtail Millet Whole Grain on Kidney Damage in a Diabetic Mouse Model
Source: Front Nutr. 2022 Jun 16;9:940404. doi: 10.3389/fnut.2022.940404 (PMC9243661; doi:10.3389/fnut.2022.940404)

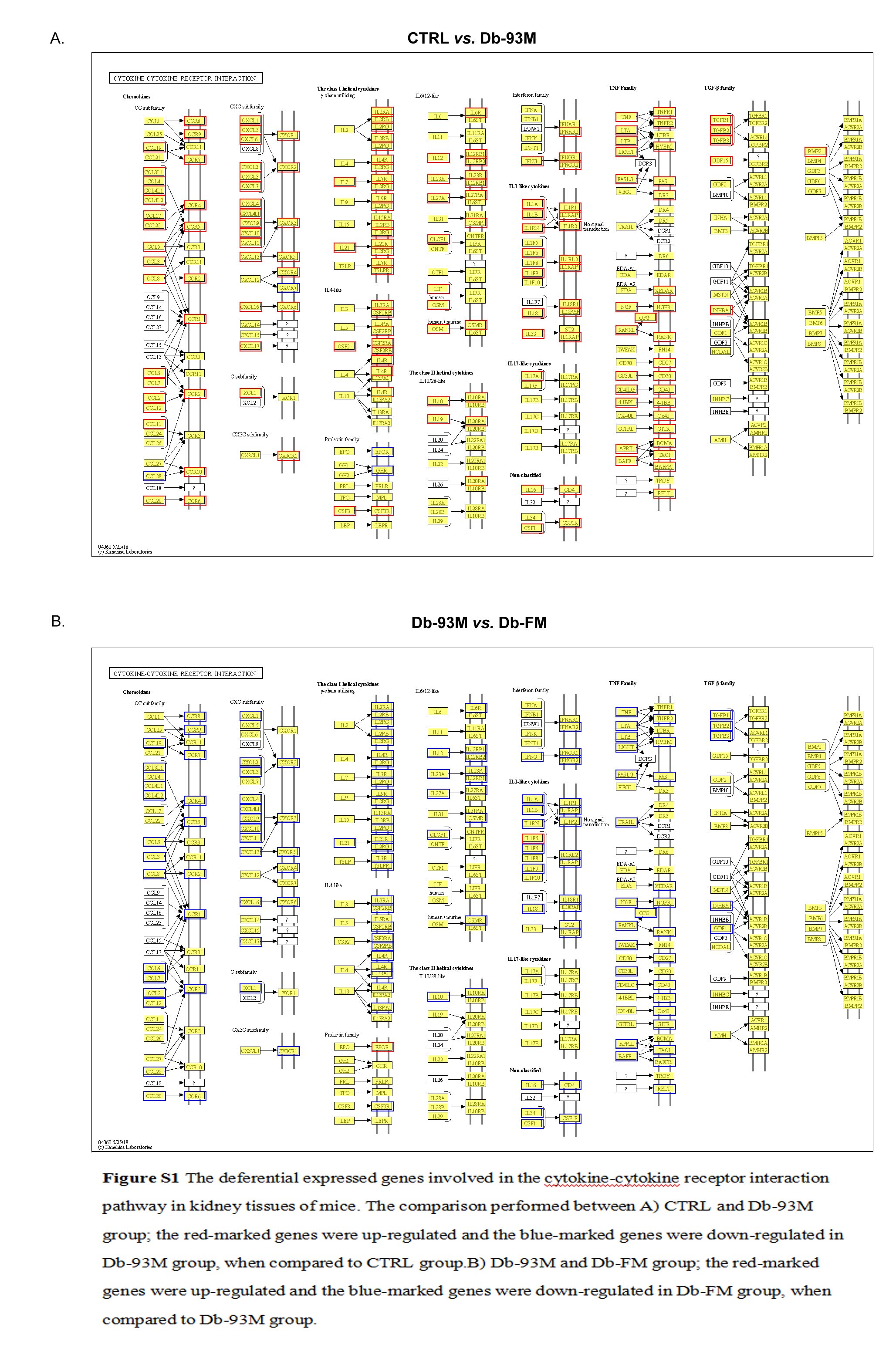

Supplement: Supplementary file 1 [file Data_Sheet_1.ZIP › Figure S1.jpg]

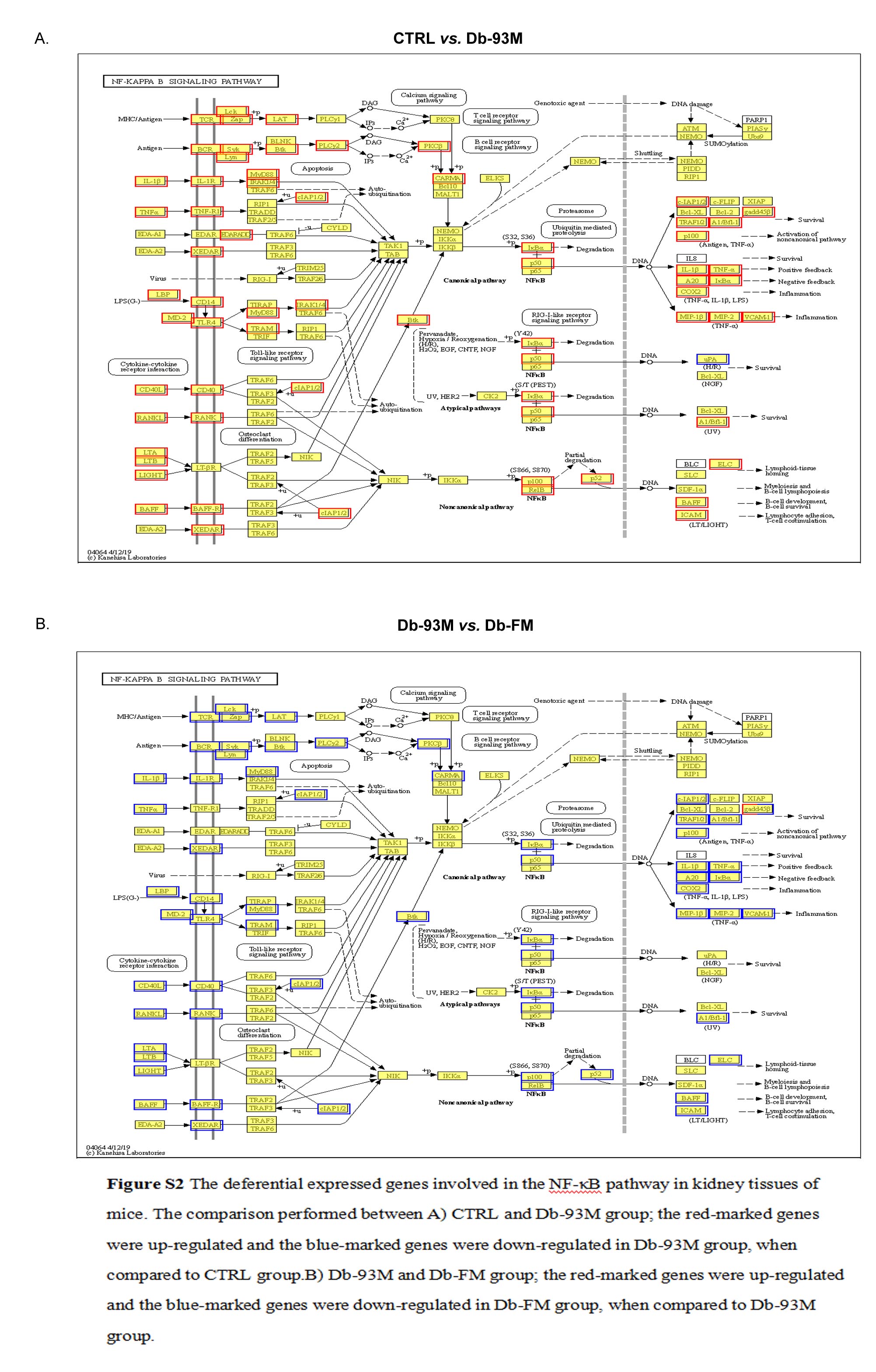

Supplement: Supplementary file 1 [file Data_Sheet_1.ZIP › Figure S2.jpg]

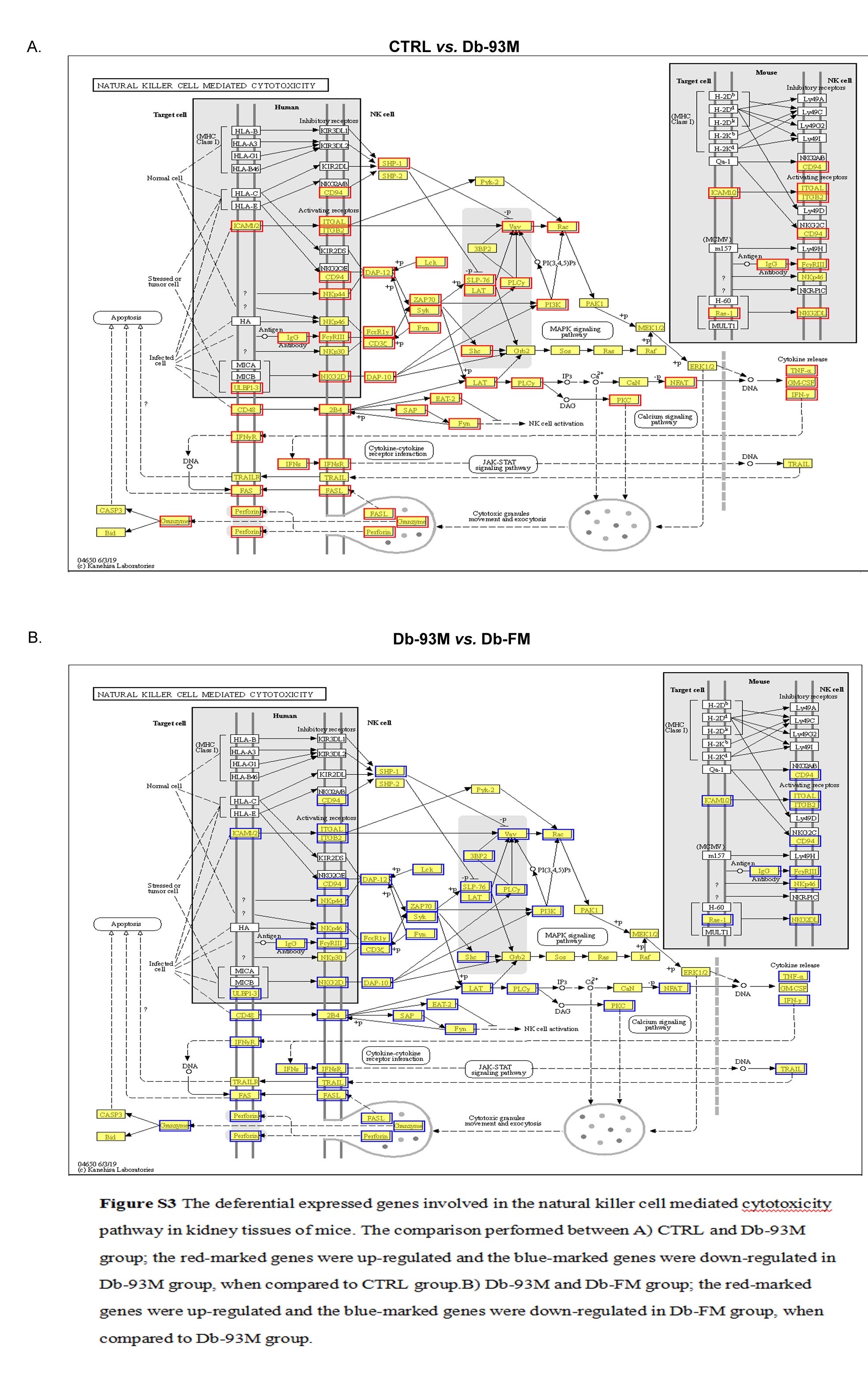

Supplement: Supplementary file 1 [file Data_Sheet_1.ZIP › Figure S3.jpg]

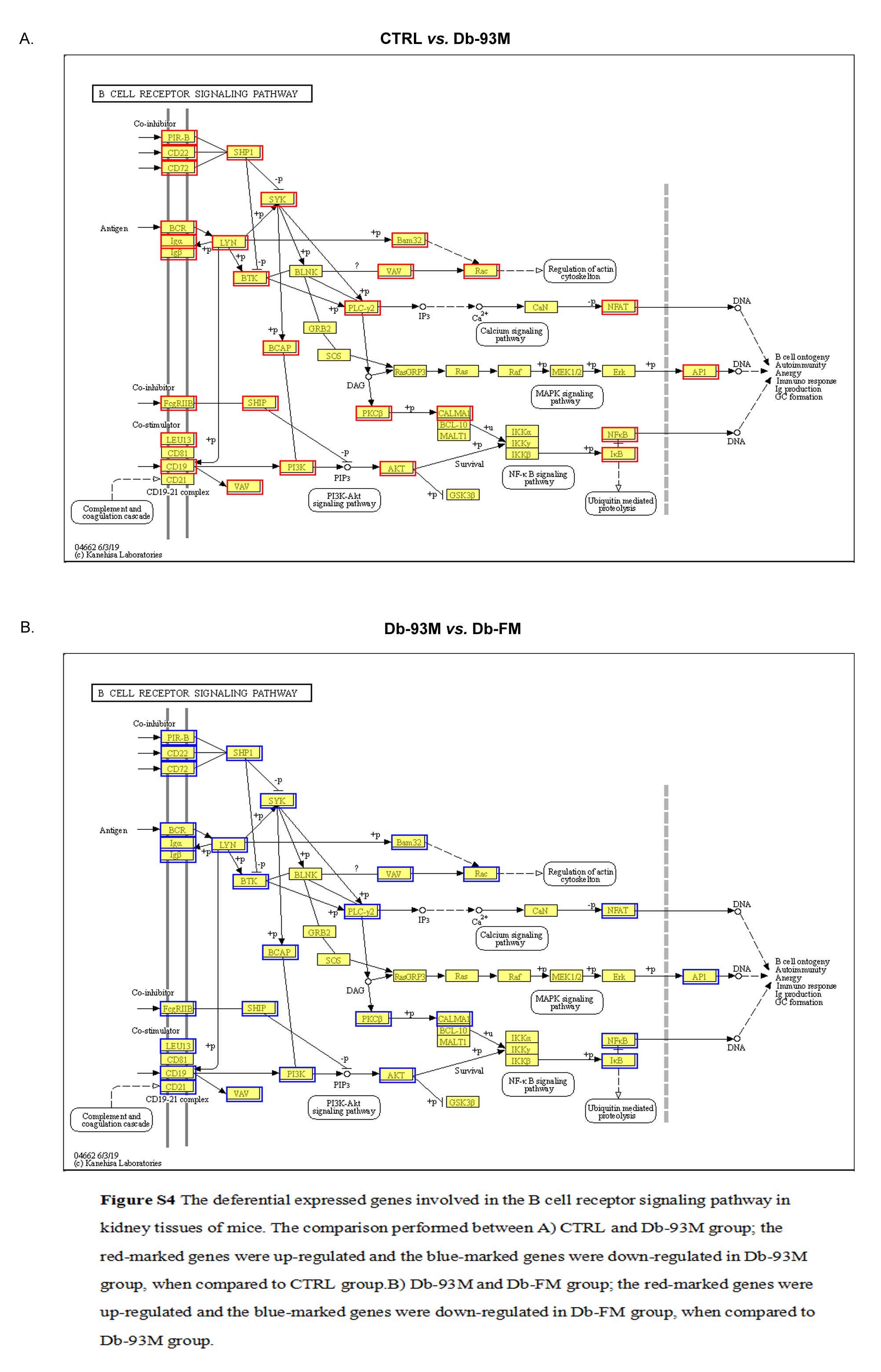

Supplement: Supplementary file 1 [file Data_Sheet_1.ZIP › Figure S4.jpg]

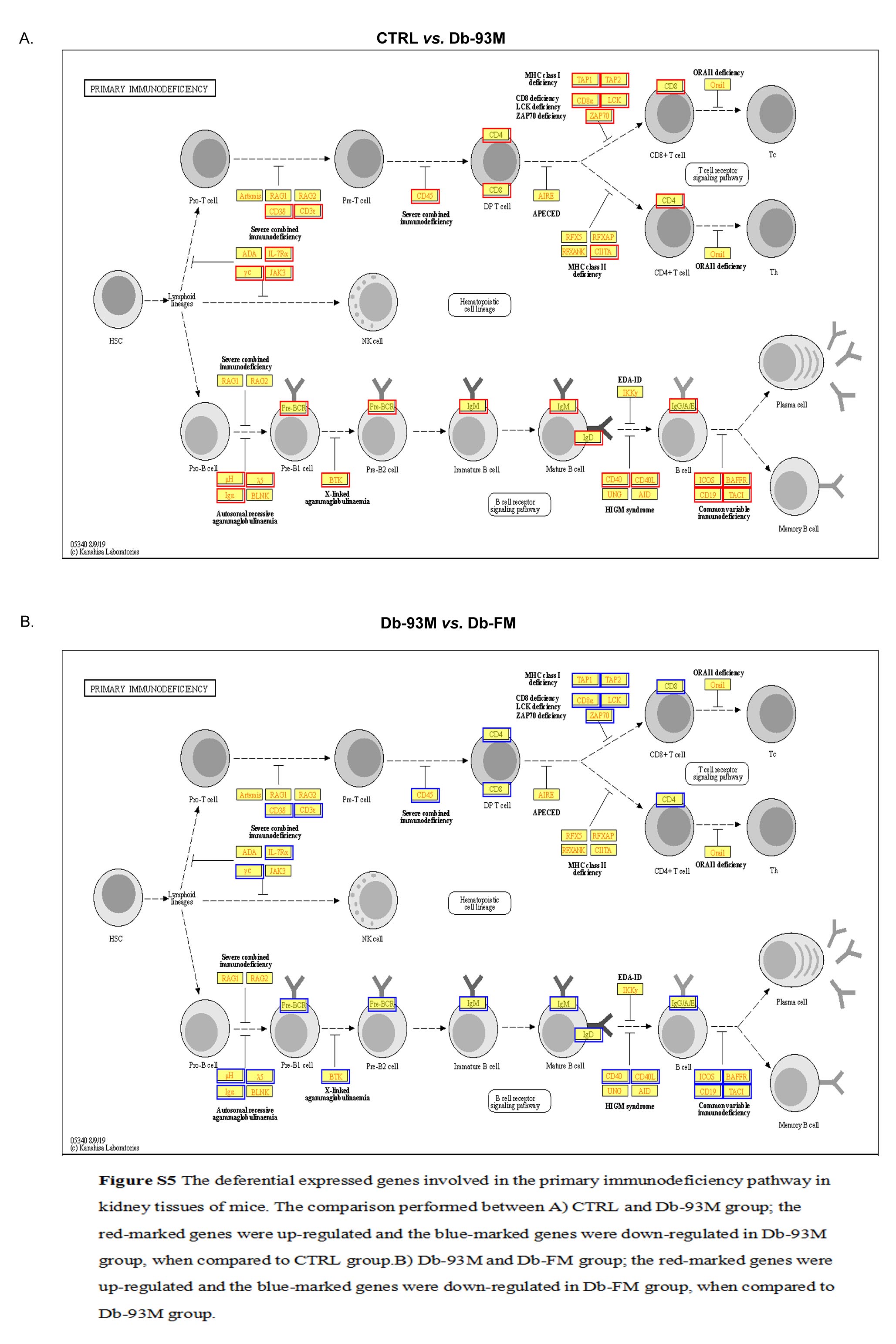

Supplement: Supplementary file 1 [file Data_Sheet_1.ZIP › Figure S5.jpg]
